# The Potential Use of Layer Litter in Awassi Lamb Diet: Its Effects on Carcass Characteristics and Meat Quality

**DOI:** 10.3390/ani9100782

**Published:** 2019-10-11

**Authors:** Belal S. Obeidat, Mohammad A. Mayyas, Abdullah Y. Abdullah, Mofleh S. Awawdeh, Rasha I. Qudsieh, Mohammad D. Obeidat, Basheer M. Nusairat, Kamel Z. Mahmoud, Serhan G. Haddad, Fatima A. Al-Lataifeh, Mysaa Ata, Majdi A. Abu Ishmais, Ahmed E. Aljamal

**Affiliations:** 1 Department of Animal Production, Faculty of Agriculture, Jordan University of Science and Technology, Irbid 22110, Jordan; 2 Department of Veterinary Pathology and Public Health, Faculty of Veterinary Medicine, Jordan University of Science and Technology, Irbid 22110, Jordan; 3 Prestage Department of Poultry Science, North Carolina State University, Raleigh, NC 27695-7608, USA; 4 Department of Animal Production and Protection, Faculty of Agriculture, Jerash University, Jerash 26150, Jordan

**Keywords:** awassi lambs, carcass characteristics, meat quality, layer litter

## Abstract

**Simple Summary:**

Inclusion of local agro–industrial by-products as alternatives to cereal-based concentrates is a promising solution with an increased usage in the area. In this study layer litter is included at 0, 150, or 300 g/kg in the diets of growing lambs. Except with minor effects on carcass characteristics for lambs fed layer liter at 150 g/kg, the inclusion of layer liter did not affect carcass characteristics and meat quality.

**Abstract:**

Carcass parameters and meat quality in lambs that consumed diets having layer hen litter (LL) were evaluated in a complete randomized study. Forty-two lambs were allocated equally (14 lambs/treatment diet) into one of three iso-nitrogenous diets for 75 days. To partially replace soybean meal and barley, LL was given at 0 (LL0), 150 (LL150), or 300 g/kg (LL300) of dietary dry matter (DM). At the termination of the trial, the characteristics of carcasses (hot and cold carcass weight, dressing percentage, and carcass cuts) and meat quality (*Musculus longissimus linear dimensions*, ultimate pH, cooking loss, water holding capacity (WHC), shear force (SF), color coordinates) were measured after slaughtering all lambs. *Longissimus* muscle weight was greatest (*p* < 0.05) for the LL150. For the dissected loin, intermuscular fat content was lowest for the LL0 diet. However, subcutaneous fat content was lower (*p* < 0.05) in the LL300 diet than LL0 and LL150 diets. Rib fat depth and *Musculus longissimus* area were greater (*p* < 0.05) for LL150 than L0. No differences were found in meat pH or color parameters among treatments but WHC and SF were lower in L0 lambs than in lambs fed LL containing diets. Cooking loss was greater for the LL300 diet than the LL0 diet. In summary, quality of meat and carcasses data indicate the possibility of inclusion of LL up to 300 g/kg DM to growing Awassi lambs.

## 1. Introduction 

A good approach to measure the productivity of slaughtering animals is by estimating the efficiency of production. Almost 70% of the cost of red meat production is associated with diet ingredients, which alerts the necessity to use unconventional feed ingredients in order to mitigate the production cost [1]. Furthermore, feed sources available for ruminants during the dry season are of low quality and have low protein content in Jordan and worldwide [2]. Therefore, layer hen litter (LL) is a possible feed to be used as an alternative feed ingredient to provide protein for ruminants [3]. Noland et al. [4] were among the first to investigate the potential for using broiler litter as a nitrogen source for sheep. Sheep have the ability to digest this cheap nitrogen-rich LL and, at the same time, poultry producers would have a safe way for disposing LL. Several trials have reported successful performance with inclusion of LL in sheep rations [5,6,7]. For example, Obeidat et al. [6] reported that growth performance, carcass characteristics, and meat quality was not affected when broiler litter was included in the diets of lambs at 0, 100, or 200 g/kg dry matter (DM). However, the effects of feeding LL on characteristics of carcasses and quality of meat are still essential to investigate. Azizi-Shotorkhoft et al. [8] reported that feeding up to 210 g/kg dry matter of heat-processed broiler litter to fat-tailed Moghani lambs reduced loin fat weights, and had no effects on lean, bone, and fat weights in carcasses.

Our hypothesis was that feeding LL to Awassi lambs during the fattening period would not impact carcass characteristics and meat quality. Therefore, this experiment was conducted to evaluate the influence of the inclusion of different proportions of LL on carcass and meat quality parameters of growing Awassi lambs.

## 2. Materials and Methods 

### 2.1. Experimental Procedures

The Institutional Animal Care and Use Committee approved all methods and procedures used in the current study at Jordan University of Science and Technology (JUST). Procedures and data of nutrient intake and growth performance were previously described in Obeidat et al. [9]. In brief, forty-two Awassi lambs (20.5 ± 0.88 kg initial body weight (BW), 70 ± 2.02 days of age) were assigned randomly into one of three iso-nitrogenous [174 g/kg crude protein (CP); dry matter (DM) basis] diets. To partially replace soybean meal and barley, LL was given at 0 (LL0), 150 (LL150), or 300 g/kg (LL300) of dietary DM. Lambs were individually housed (1.5 × 0.75 m) and the experimental diets were offered twice daily ad libitum during the whole experimental period (75 days). The feed intake was calculated by subtracting the collected refusal from the offered feed. In brief, DM intake, final body weight, and average daily gain were greater in lambs fed diets containing LL at 150 g/kg DM compared with the lambs fed LL at 0 g/kg DM, whereas in lambs fed LL at 300 g/kg DM, results were intermediate. Dry matter intake was 894, 1006, and 940 g/d for LL0, LL150, and LL300 g/kg, respectively. Layer litter was obtained from a local floor-reared laying hen farm. Before mixing the diets, LL was placed in plastic bags and autoclaved at 121 °C for 20 min to kill litter microflora. After autoclaving, LL was ground to pass a 3 mm screen in order to facilitate its mixing with the other dietary ingredients (Table 1). The chemical composition of the LL and the diet ingredients was analyzed following the procedures of AOAC [10]. Composition of the LL and experimental diets is shown in Table 1.

### 2.2. Slaughtering Procedures and Meat Quality Measurements

At the end of the experimental period, lambs were transported to a slaughterhouse. After 18 h of fasting, live BW was recorded and animals were slaughtered following the procedure described by Abdullah et al. [11]. Non-carcass components (spleen, liver, kidneys, lungs, and trachea) were removed and weighed and carcass weight was recorded immediately after slaughter (HCW) and after 24 h of refrigeration at 4 °C (CCW) to calculate chilling losses and dressing percentage. Upon cutting, the right leg and loin cut were dissected and vacuum-packed immediately and stored at −20 °C for further measurements. Then, the *longissimus* muscle was removed and kept frozen at −20 °C for further analysis. 

To measure meat quality parameters of the *longissimus* muscle, frozen muscle was thawed over night at 4 °C using the procedure described by Obeidat et al. [12]. The measured parameters were water holding capacity (WHC), meat color, pH, shear force, and cooking loss. In brief, each thawed muscle was divided into slices of 15 mm thick to be used to measure color coordinates (CIE L*a*b*). To allow oxygenation of the sample, 2 h prior to the measurement, color slices were covered with permeable film and kept at 4 °C. Then, color coordinates were measured in triplicates by using a handheld colorimeter device (12 mm Aperture U 59730-30, Cole-Parameter International, Accuracy Microsensors Inc., Pittsford, NY, USA). Cooking loss was measured using duplicate-slices (25 mm thickness) after weighing raw slices and placing and cooking them in plastic bags in a water bath at 75 °C for 90 min (until reaching an internal temperature of 72 °C). After bathing the slices, weight was measured to calculate water lost percentage. To measure the shear force (i.e., tenderness measurement) cooked slices were then stored in the chiller for 24 h at 4 °C. By using a Warner–Bratzler (WB) shear blade (Warner-Bratzler meat shear, GR manufacturing Co. 1317 Collins LN, Manhattan, Kansas, 66502, USA) with the triangular slot cutting edge mounted on a Salter Model 235, a total of 6 cores, with a size of 1 cm^3^, were cut from the slices and sheared in a perpendicular direction of muscle fiber. Peak force (kg) required to shear the cores was measured as an indicator of meat tenderness. Muscle pH was evaluated on a homogenate of 2 g of raw meat and 10 mL of neutralized 5 mM iodoacetate reagent using a pH spear (pH Spear, Eutech Instrument, USA). Water holding capacity was evaluated using methods described by Grau and Hamm [13]. 

### 2.3. Statistical Analysis

Data were subjected to analysis of variance using the mixed procedure of SAS (Version 8.1, 2000, SAS Inst. Inc., Cary, NC, USA). [14]. Diet was included as fixed effect in the statistical model, the animal nested to diet being the residual error. Mean separation for all traits was conducted using pair-wise *t*-tests and significance level was determined at *p* < 0.05.

## 3. Results 

Fasting weight, HCW, CCW, dressing percentage, and non-carcass components were not significantly different (*p* > 0.05) among treatment diets (Table 2). However, carcass cut weights were greater (*p* < 0.05) in lambs fed the LL150 diet than the LL0 and LL300 diets. Fat tail and mesenteric fat were similar (*p* > 0.05) among the diets. The weight of kidney fat was greater (*p* < 0.05) in the LL150 diet than the LL0 diet, whereas the LL300 was intermediate.

Results showed that lambs fed LL150 diet had greater (*p* < 0.05) weight of loin cut compared with lambs fed the LL0 diet (Table 2). *Longissimus* muscle was greater (*p* < 0.05) for the LL diets than the control group (LL0). The content of intermuscular fat was lower (*p* < 0.05) for lambs fed the LL0 than for lambs fed the LL150. Subcutaneous fat content was lower (*p* < 0.05) in the LL300 than the LL0 and LL150 diets. Content of total lean and fat and ratio of meat to fat did not differ (*p* > 0.05) among diets. However, total bone content was higher (*p* < 0.05) in lambs fed LL300 than LL0 or LL150 diets. As a result, the ratio of meat to bone was lower (*p* < 0.05) in LL300 diets than the LL0 and LL150 diets.

Intermuscular fat content was lower (*p* < 0.05) in the LL300 diet than the LL150 diet, while the LL0 diet was similar compared with LL150 and LL300 diets in the dissected leg (Table 2). However, the content of total fat and subcutaneous fat were greater significantly in LL150 compared with the LL0 and LL300 diets. However, the content of total bone and lean was comparable among different diets. The ratio of meat to bone in the loin was lower (*p* < 0.05) in lambs fed the LL300 than lambs fed the LL150 diet. However, the ratio of meat to fat in the leg was lower (*p* < 0.05) in the LL150 diet compared to the LL0 and LL300 diets. Meat to bone ratio in the leg was lower (*p* < 0.05) in the LL150 diet than the LL0 and LL300 diets. Tissue depth did not differ among diets. However, the depth of rib fat was greater (*p* < 0.05) in lambs fed the LL150 diet compared to lambs fed the LL0 and LL300 diets. No significant difference was observed for the shoulder fat depth between the diets.

Results of meat quality are shown in Table 3. Ultimate pH did not differ (*p* > 0.05) among diets. Cooking loss was greater (*p* < 0.05) in lambs fed the LL300 diet compared to the LL0 diet, whereas cooking loss for the LL150 diet was intermediate. However, greater water holding capacity and shear forces were observed in lambs fed LL-containing diets compared with the control diet. Meat color (i.e., *L* *, *a* *, and *b* *) did not differ among diets. *M. longissimus* width was greater (*p* < 0.05) in the LL300 diet than the LL0 diet; whereas the LL150 diet was not different from the other two diets. *M. longissimus* depth was lower (*p* < 0.05) for the LL0 diet than for the LL150 and LL300 diets. *M. longissimus* area was greater (*p* < 0.05) for the LL150 diet than for the LL0 diet. 

## 4. Discussion

In accordance with previous studies [6,15], neither carcass weight nor dressing percentage were affected by broiler litter supplementation when fed at 100 and 200 g/kg of dietary DM fed to Awassi lambs [6] and 0, 280, 560, and 850 g/kg DM) in diets of South African Mutton Merino wethers [15], which would be in agreement with results obtained herein. Nevertheless, meat to bone ratio decreased in the highest LL supplementation. Obeidat et al. [6] did not observe any effect of meat to bone ratio in leg cuts, but they tested levels of LL lower than 200 g/kg feed. In our experiment, mineral content was not equilibrated, and it increased as the LL proportion increased. Therefore, it would be possible that a greater mineral supply affects bone development or density.

When looking at the meat to fat ratio in both loin and leg cuts, diets formulated with 150 g/kg LL had the lowest ratio in both cuts (even though differences did not reach a significant level in the loin) indicating that total fat content was highest in those lambs. It is the metabolic rate and physiological importance that play an important role in the process of separating the use of nutrients between tissues and different organs in the body. Therefore, when providing ad libitum balanced feed, the highest efficiency of feed conversion was achieved. Based on live performance data of Obeidat et al. [9], lambs fed LL150 had the highest dry matter intake, final body weight, and the best feed efficiency compared to the other treatments. Dry matter intake was 894, 1006, and 940 g/d for LL0, LL150, and LL300 g/kg, respectively.

Consistent with differences observed among treatments in carcass cuts, LL150 lambs showed significantly greater rib fat depth and kidney fat, mesenteric fat and shoulder fat depth being numerically greater as well. In a study that compared linear dimensions of *M. longissimus* and measurements of fat for lambs that consumed broiler litter at various levels (i.e., 0, 100, or 200 g/kg), Obeidat et al. [6] reported that tissue depth, *M. longissimus* depth, width, and area, fat depth, fat tail weight, and weight of kidney fat did not change among dietary treatments. On the other hand, rib fat depth or mesenteric fat weight were lower for 200 g/kg broiler litter diet compared with the control diet but 100 g/kg broiler litter diet did not differ from the other two treatments [6]. Furthermore, *M. longissimus* dimensions were also the largest in lambs fed 150 g LL/ kg feed, which is in accordance with the heaviest weight of *M. longissimus* harvested from those lambs. The discrepancies in linear dimensions that was observed among different studies could be due to the difference in the diets’ compositions, the chemical content in the LL, and/or the age and the type of animals. 

Values for meat quality attributes measured in the current study were within acceptable range. Consistent with our results, Obeidat et al. [6]) reported that broiler litter supplementation at 0, 100, or 200 g/kg DM did not affect the pH of meat. Ultimate pH of *M. longissimus* observed herein did not differ from other studies [16,17,18]. Inconsistent with results obtained herein, some of the meat quality characteristics (shear force, cooking loss, or water holding capacity) was similar when lambs fed diets containing various levels of broiler litter (0, 100, and 200 g/kg of dietary DM) [6]. In addition, shear force was not different in Holstein steers fed diets containing broiler litter [19]. In the current study, shear force values were higher for *M. longissimus* from LL-fed lambs, which relates to higher cooking loss, and thus the meat was drier. Despite that, the shear force values were greater in diets containing LL when compared to the control diet; they were still within the normal range. Color coordinates measured were comparable among the different diets. Overall, current results showed that feeding LL to Awassi lambs did not negatively affect meat quality except for the greater shear force in diets containing LL.

## 5. Conclusions

Results of the current study shows that the inclusion of layer litter at 0, 150, or 300 g/kg of DM did not affect fasting live weight, hot and cold carcass weight, and dressing percentage among diets. However, there are few minor changes in the meat quality parameters especially in lambs fed the 150 g/kg diet. Therefore, it is recommended to feed diet containing layer litter between 150 to 300 g/kg to lambs.

## Figures and Tables

**Table 1:** Ingredients and chemical composition of diets and layer litter (LL) fed to Awassi lambs.

Item	Diets ^1^			
	LL0	LL150	LL300	LL
Ingredients (g/kg DM)				
Barley grain	629	519	409	
Soybean meal (440 g/kg CP ^2^ (solvent))	150	110	70	
Layer litter	0	150	300	
Wheat straw	200	200	200	
Soybean oil	5	5	5	
Salt	7.5	7.5	7.5	
Limestone	7.5	7.5	7.5	
Mineral vitamin premix ^3^	1.0	1.0	1.0	
Nutrients				
Dry matter (g/kg DM)	909	907	916	895
Organic matter (g/kg DM)	926	890	878	725
Crude protein (g/kg DM)	173	174	174	269
Neutral detergent fiber (g/kg DM)	226	254	274	236
Acid detergent fiber (g/kg DM)	117	142	154	97
Ether extract (g/kg DM)	69	66	65	25
Copper (µg/g)	4.86	8.76	14.18	41.6

^1^ Diets were: LL included in the diets at 0 (LL0), 150 (LL150), and 300 g/kg (LL300) of dietary dry matter (DM). ^2^ CP: crude protein, ^3^ Composition per kg use (vitamin A, 2,000,000 IU; vitamin D_3_, 40,000 IU; vitamin E, 400 mg, Mn, 12.80 g; Zn, 9.00 g; I, 1.56 g; Fe, 6.42 g; Cu, 1.60 g; Co, 50 mg; Se, 32 mg).

**Table 2:** Carcass components, dissected loin and leg carcass cut weights, and percentages and fat depth of Awassi lambs fed diets containing layer litter.

Item	Diets ^1^			
	LL0	LL150	LL300	SEM ^2^
Fasting live weight (kg)	35.7	38.4	36.6	1.32
Hot carcass weight (kg)	16.7	18.2	16.8	0.72
Cold carcass weight (kg)	16.1	17.6	16.1	0.70
Dressing percentage (%)	45.7	45.8	44.3	1.19
Non-carcass components (kg ^3^)	1.42	1.41	1.33	0.06
Carcass cut weights (kg ^4^)	14.4 ^a^	16.3 ^b^	15.3 ^a^	0.46
Fat tail (kg)	1.60	1.71	1.42	0.16
Mesenteric fat (g)	348	407	327	45.0
Kidney fat (g)	157 ^a^	228 ^b^	185 ^ab^	20.1
Loin weight (g)	807 ^a^	976 ^b^	879 ^ab^	39.8
Longissimus muscle (g)	200.7 ^a^	252.1 ^b^	237.9 ^b^	8.01
Intermuscular fat (g/100 g)	5.70 ^a^	7.13 ^b^	6.71 ^ab^	0.53
Subcutaneous fat (g/100 g)	16.9 ^b^	16.7 ^b^	12.8 ^a^	1.29
Total fat (g/100 g)	22.6	23.9	19.5	1.64
Total lean (g/100 g)	51.1	49.7	50.9	1.30
Total bone (g/100 g)	17.7 ^a^	17.9 ^a^	22.4 ^b^	0.77
Meat to bone ratio	2.93 ^b^	2.87 ^b^	2.30 ^a^	0.10
Meat to fat ratio	2.85	2.22	2.89	0.272
Leg weight (g)	2675	2937	2891	108.3
Intermuscular fat (g/100 g)	3.21 ^ab^	3.50 ^b^	3.00 ^a^	0.17
Subcutaneous fat (g/100 g)	13.9 ^a^	16.6 ^b^	13.7 ^a^	0.62
Total fat (g/100 g)	17.0 ^a^	20.1 ^b^	16.6 ^a^	0.66
Total lean (g/100 g)	58.3	59.3	57.9	1.65
Total bone (g/100 g)	19.2	19.3	19.7	0.62
Meat to bone ratio	3.05 ^ab^	3.10 ^b^	2.95 ^a^	0.050
Meat to fat ratio	3.57 ^b^	3.02 ^a^	3.52 ^b^	0.15
Tissue depth (GR) (mm)	14.9	16.2	14.3	0.91
Rib fat depth (J) (mm)	5.90 ^a^	8.62 ^b^	5.81 ^a^	0.64
Shoulder fat depth (C) (mm)	3.92	4.71	3.60	0.61

^1^ Diets were: layer litter (LL) included in the diets at 0 (LL0), 150 (LL150), and 300 g/kg (LL300) of dietary dry matter. ^2^ SEM: Standard Error of the Mean, ^3^ Non-carcass components (heart, liver, spleen, kidney, and lungs and trachea). ^4^ Carcass cut (shoulder, racks, loins, and legs). ^ab^ Within a row, means without a common superscript differ (*p* < 0.05).

**Table 3:** Meat quality characteristics and *M. longissimus* linear dimensions of Awassi lambs fed finishing diets containing layer litter.

Item	Diets ^1^			
	LL0	LL150	LL300	SEM ^2^
Ultimate pH ^3^	5.87	5.89	5.84	0.019
Cooking loss (g/100 g)	41.6 ^a^	42.6 ^ab^	43.2 ^b^	0.61
Water holding capacity (g/100)	21.6 ^a^	24.6 ^b^	24.2 ^b^	0.73
Shear force (kg/cm^2^)	5.8 ^a^	8.8 ^b^	7.7 ^b^	0.50
Color coordinates				
L * (whiteness)	38.9	38.3	39.4	0.50
a * (redness)	3.0	2.8	3.7	0.54
b * (yellowness)	19.0	18.8	19.3	0.63
*M. longissimus* width (A) (mm)	26.1 ^a^	27.3 ^ab^	28.2 ^b^	0.70
*M. longissimus* depth (B) (mm)	55.1 ^a^	59.9 ^b^	58.1 ^b^	0.81
*M. longissimus* area (cm^2^)	13.4 ^a^	15.3 ^b^	14.3 ^ab^	0.35

^1^ Diets were: layer litter (LL) included in the diets at 0 (LL0), 150 (LL150), and 300 g/kg (LL300) of dietary dry matter. ^2^ SEM: Standard Error of the Mean, ^3^ pH measured after thawing. ^ab^ Within a row, means without common letters differ (*p* < 0.05).
